# Characterization of Silver Nanowire-Based Transparent Electrodes Obtained Using Different Drying Methods

**DOI:** 10.3390/nano12030461

**Published:** 2022-01-28

**Authors:** Seo Bum Chu, Dongwook Ko, Jinwook Jung, Sungjin Jo, Dong Choon Hyun, Hyeon-Ju Oh, Jongbok Kim

**Affiliations:** 1 Department of Materials Science and Engineering, Kumoh National Institute of Technology, Gumi 39177, Korea; csb5453@naver.com (S.B.C.); duko1293@gmail.com (D.K.); jkim3161@gmail.com (J.J.); 2 School of Architectural, Civil, Environmental, and Energy Engineering, Kyungpook National University, Daegu 41566, Korea; sungjin@knu.ac.kr; 3 Department of Polymer Science and Engineering, Kyungpook National University, Daegu 41566, Korea; dong.hyun@knu.ac.kr; 4 Advanced Materials Research Center, Kumoh National Institute of Technology, Gumi 39177, Korea; 5 Department of Energy Engineering Convergence, Kumoh National Institute of Technology, Gumi 39177, Korea

**Keywords:** silver nanowire, transparent electrode, drying method, electrical property, morphology

## Abstract

Metal-based transparent top electrodes allow electronic devices to achieve transparency, thereby expanding their application range. Silver nanowire (AgNW)-based transparent electrodes can function as transparent top electrodes, owing to their excellent conductivity and transmittance. However, they require a high-temperature drying process, which damages the bottom functional layers. Here, we fabricated two types of AgNW-based electrodes using the following three drying methods: thermal, room-temperature, and vacuum. Thereafter, we investigated the variation in their morphological, electrical, and optical characteristics as a function of the drying method and duration. When the AgNW-exposed electrode was dried at room temperature, it exhibited a high surface roughness and low conductivity, owing to the slow solvent evaporation. However, under vacuum, it exhibited a similar electrical conductivity to that achieved by thermal drying because of the decreased solvent boiling point and fast solvent evaporation. Conversely, the AgNW-embedded electrodes exhibited similar roughness values and electrical conductivities regardless of the drying method applied. This was because the polymer shrinkage during the AgNW embedding process generated capillary force and improved the interconnectivity between the nanowires. The AgNW-based electrodes exhibited similar optical properties regardless of the drying method and electrode type. This study reveals that vacuum drying can afford transparent top electrodes without damaging functional layers.

## 1. Introduction

Generally, electronic devices are fabricated by the sequential stacking of functional layers, although different materials are employed depending on the device type. For each device, many scholars investigated the effect of modifying the functional layer characteristics on the final properties of electronic devices. Additionally, numerous studies have examined the phenomena occurring at the interface between functional materials and the approach to maximize the final properties through the optimization of the entire device [1,2,3,4,5,6]. Recently, numerous researchers have attempted to fabricate functional materials in various shapes to increase their application range [7,8,9]. For example, flexible sensors that can be combined with various devices to monitor the body or surroundings in real-time, stretchable solar cells and transistors that can be attached to our skin have been investigated [10,11,12,13]. To further expand the application range of electronic devices, the transparency property is essential. For instance, if electronic devices are transparent, they can be integrated into different devices, such as displays, and installed on windows [14,15]. However, while the bottom electrode of electronic devices can achieve transparency using indium tin oxide electrodes, it is difficult for the metal top electrode to achieve transparency, which limits the application range of electronic devices.

One method for achieving a transparent metal top electrode involves fabricating the top electrode using graphene, carbon nanotubes, or metal nanowires [16,17,18,19]. In this regard, silver nanowires (AgNWs) are promising candidates because transparent bottom electrodes fabricated using AgNWs have been reported to exhibit excellent conductivity, transmittance, and flexibility [19,20] and AgNW technologies continue to evolve. For example, it has been reported that Ag nanowire-based hybrid electrodes comprising Ag nanowires, carbon nanotube, and graphene oxide can achieve efficient wearable sensors [21] and organic photovoltaics [22]. In addition, the electrospray assisted etching of Ag nanowires was able to fabricate a quasi-periodic structure with the potential for various optoelectronic applications [23] and several patterning methods for Ag nanowires including subtractive methods, additive methods and printing methods were developed to achieve a precise patterning of Ag nanowire networks [24,25]. To this end, we recently adopted AgNWs as metal top electrodes and fabricated a transparent speaker that could be attached to a display [26]. Specifically, when using a AgNW solution with a solvent to prepare the top electrode, its functional layers can be oxidized or crystallinity can be changed during the thermal drying process to evaporate the solvent, resulting in the deterioration of the device performance. We explored various methods of drying AgNWs to overcome the thermal damage, and we succeeded in producing transparent speakers. However, the differences between the AgNW-based transparent electrodes dried by thermal annealing and those dried by different methods have not yet been clarified.

In this study, we investigated the properties of AgNW-based transparent electrodes as a function of the applied drying method. Two types of transparent electrodes, AgNW-exposed electrodes and AgNW-embedded electrodes, were fabricated, and their morphological, electrical, and optical characteristics were examined. The AgNW-exposed electrodes exhibited different surface roughness values and electrical properties because the drying methods affected the rate of solvent evaporation and the degree of solvent removal. In contrast, the AgNW-embedded electrodes exhibited a comparable surface roughness and electrical resistance properties regardless of the drying method because the AgNWs were embedded in a polymer, the shrinkage of the polymer during the polymerization process generated a capillary force and enhanced the interconnectivity between the AgNWs; notably, the initial properties of the materials before the embedding process were different. The surface roughness and electrical resistance of the AgNW-embedded electrodes were lower than those of the AgNW-exposed electrodes. Conversely, their optical properties were similar regardless of the electrode type and the drying method, showing an average transmittance of approximately 83% in the visible range.

## 2. Materials and Methods

**Figure 1:**
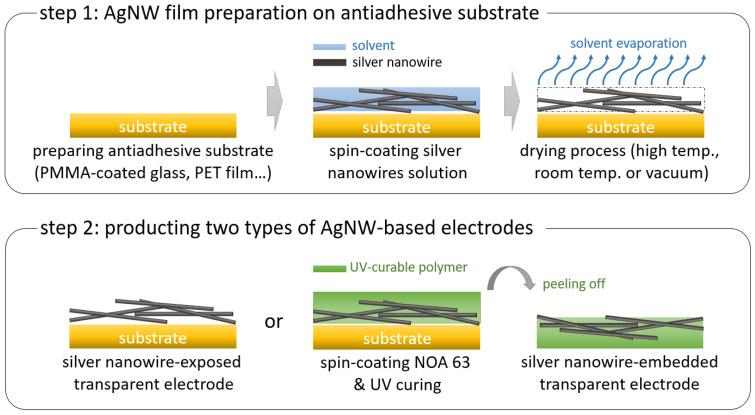
Scheme of the fabrication process of two types of silver nanowire (AgNW)-based transparent electrodes.

Two types of AgNW-based transparent electrodes were prepared using various drying processes. The fabrication process comprised the following two steps: (1) preparation of a AgNW film on a substrate and (2) completion of the electrode preparation as an exposed type or an embedded type (Figure 1). In the AgNW-exposed electrode, a poly(ethylene terephthalate) (PET) film, which is suitable for electronic devices, was employed as the substrate. After cutting the PET film to an appropriate size, the protective film was removed to secure a clean PET surface. Thereafter, the surface was spin-coated with a AgNW solution (0.3 wt% in deionized water, C3nano Korea, Yongin, Korea) at 1000 rpm for 60 s to form the AgNW film.

Since the initial AgNW films contained a large amount of solvent and exhibited poor conductivity, they were dried using three different methods, namely, thermal drying, room-temperature drying, and vacuum drying. Thermal drying was conducted at 50 °C, 80 °C, and 100 °C or 120 °C for 1 min, which are typical temperatures for fabricating silver nanowire-based electrodes to avoid Plateau-Rayleigh instability [27,28,29,30,31,32]. Room-temperature drying and vacuum drying were conducted for 5, 10, 20, and 30 min at room temperature and low pressure using a desiccator and low-pressure vacuum pump, respectively. After the drying process, the electrodes were visually observed to confirm if they were well-formed. For the AgNW-embedded electrode, a poly(methyl methacrylate) (PMMA, Microchem, Newton, MA, USA)-coated glass substrate was employed as the antiadhesive substrate. To prepare the PMMA-coated glass substrate, the PMMA layer was spin-coated on a glass substrate at 3000 rpm for 60 s and annealed at 180 °C for 30 s. Thereafter, spin-coating of a AgNW solution was conducted under the same conditions applied for the AgNW-exposed electrode. Thereafter, the AgNW film was dried by thermal annealing, room-temperature drying, and vacuum drying under the same conditions described above. Afterward, the prepolymer of Norland Optical Adhesive 63 (NOA 63, Norland Products Inc., Jamesburg, NJ, USA) was spin-coated at 500 rpm for 60 s. Ultraviolet (UV) curing was applied for 60 min, and the NOA 63 film was peeled off to afford the AgNW-embedded electrodes. Full curing of NOA 63 film was confirmed by infrared spectroscopy.

After preparing the AgNW-exposed and AgNW-embedded electrodes, their morphological, electrical, and optical characteristics were investigated. The morphological properties were examined by field emission scanning electron microscopy (FE-SEM, JEOL JSM-6500F, Tokyo, Japan) and atomic force microscopy (AFM, XE-100, Park Systems, Suwon, Korea). The electrical and optical properties were measured using a 4-point probe (RC2175, EDTM Inc., Toledo, OH, USA) and by ultraviolet–visible (UV–Vis) spectroscopy (UV-2600, Shimadzu, Kyoto, Japan), respectively.

## 3. Results and Discussion

### 3.1. Morphological Characteristics

**Figure 2:**
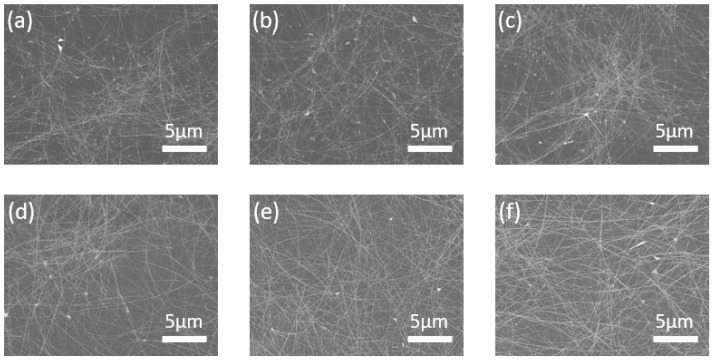
Scanning electron microscopy (SEM) images of (**a**–**c**) the AgNW-exposed electrodes and (**d**–**f**) AgNW-embedded electrodes with different drying methods: (**a**,**d**) thermal drying at 120 °C for 1 min, (**b**,**e**) room-temperature drying for 30 min, and (**c**,**f**) vacuum drying for 30 min.

To examine whether the AgNW-based electrodes were well-formed regardless of the drying process, the surfaces of the electrodes were observed by FE-SEM. Figure 2 shows the SEM images of the AgNW-exposed and AgNW-embedded electrodes dried by the three methods. Thermal drying was conducted at 120 °C for 1 min, while room-temperature drying and vacuum drying were conducted for 30 min. First, the surface images of the AgNW-exposed electrodes were similar, regardless of the drying method, as shown in Figure 2a–c. Evidently, a network of AgNWs for electrical conduction was well-formed, and the void space between the networks was sufficient to allow for optical transmittance. Since the AgNWs were coated on the substrate, the NWs were observed to protrude; however, the difference in the surface roughness as a function of the drying method was difficult to distinguish in the SEM images.

The AgNW-embedded electrodes also exhibited a well-formed AgNW network and good optical space for light transmission, as shown in Figure 2d–f. In addition, because the AgNWs were embedded in the polymer, it was observed that the NWs were covered with the polymer. However, because FE-SEM does not provide height information, it was also difficult to observe the difference in the AgNW-embedded electrodes as a function of the drying method.

**Figure 3:**
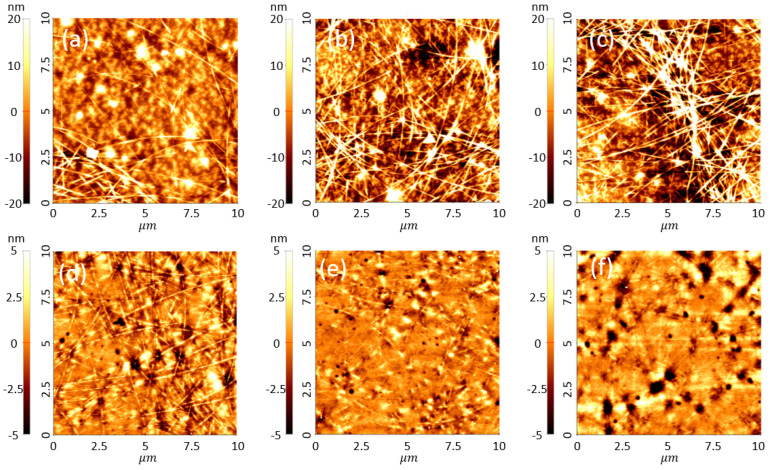
Atomic force microscopy (AFM) images of (**a**–**c**) the AgNW-exposed electrodes and (**d**–**f**) the AgNW-embedded electrodes obtained by different drying methods: (**a**,**d**) thermal drying at 120 °C for 1 min, (**b**,**e**) room-temperature drying for 30 min, and (**c**,**f**) vacuum drying for 30 min.

To observe the surface morphology in more detail, an AFM measurement was conducted. Figure 3 shows the AFM images of the AgNW-exposed and AgNW-embedded electrodes. The drying methods and conditions were the same as those described above. First, for the AgNW-exposed electrodes (Figure 3a–c), the room-temperature-dried and vacuum-dried electrodes demonstrated a higher contrast than those dried by thermal annealing, indicating that they have rougher surfaces. Specifically, the root mean square (RMS) roughness of the thermally dried electrode was 5.96 nm, while those of the electrodes subjected to room-temperature drying and vacuum drying were 8.21 and 9.89 nm, respectively. Thermal drying provided a sufficient driving force for solvent evaporation and the rearrangement of the AgNWs since it was conducted at a high temperature. Thus, thermal drying is effective for the AgNWs, and it generates capillary force during the solvent evaporation, enhancing the interconnectivity between the NWs and affording a relatively smooth surface. However, for room-temperature drying, since the room temperature is below the boiling point of the solvent, the solvent evaporation is challenging, and there is no thermal driving force to rearrange the NWs, resulting in rough surfaces. For the vacuum-drying method, the drying pressure is lower than the atmospheric pressure; thus, the solvent can evaporate, even at low temperatures. However, there is no driving force to rearrange the NWs, which results in rough surfaces. Conversely, the AgNW-embedded electrodes showed similar surface roughness values regardless of the drying method applied. The RMS roughness values were 1.23, 1.20, and 1.40 nm for the thermal drying, room-temperature drying, and vacuum drying methods, respectively. The AgNW-embedded electrode was fabricated by coating AgNWs on a substrate, drying the product, and coating/curing the polymer; resultantly, all the AgNWs were inside the polymer, and only the NWs at the bottom of the antiadhesive substrate were exposed to the surface. Therefore, although the surface roughness values of the AgNWs on the antiadhesive substrate were different, the roughness values of the three different AgNW-embedded electrodes were similar, regardless of the drying method. Resultantly, the AgNW-exposed electrodes exhibited different surface morphologies depending on the drying method. However, the AgNW-embedded electrode did not exhibit morphological differences, suggesting that various drying methods can be applied to improve the properties of transparent electronic devices.

### 3.2. Electrical Characteristics

**Figure 4:**
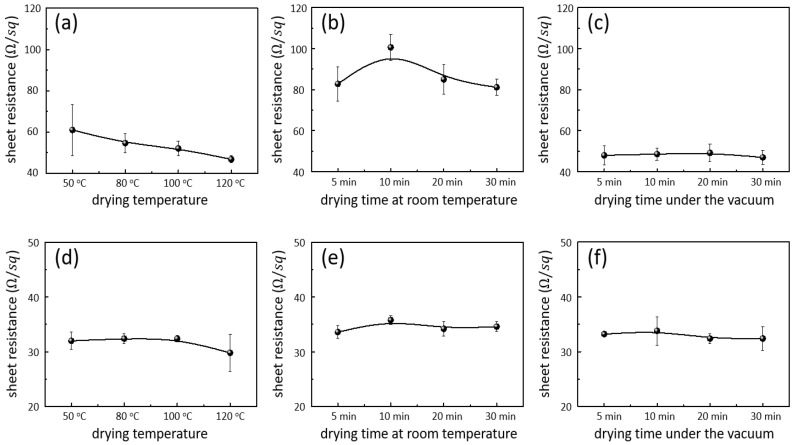
Sheet resistance of (**a**–**c**) the AgNW-exposed electrodes and (**d**–**f**) AgNW-embedded electrodes obtained using the following drying methods against the drying temperature or drying time: (**a**,**d**) thermal drying, (**b**,**e**) room-temperature drying, and (**c**,**f**) vacuum drying.

Figure 4 shows the sheet resistances of the AgNW-exposed and AgNW-embedded electrodes, which were dried by thermal, room-temperature, and vacuum methods. The drying temperature range for thermal drying was 50–120 °C, and the drying time was 1 min. The durations for the room-temperature drying and vacuum drying ranged from 5 to 30 min. First, for thermal drying, the sheet resistance decreased from 61 ± 12 Ω/sq to 47 ± 2 Ω/sq as the drying temperature increased (Figure 4a). When the AgNW solution was coated on the substrate, the solvent remained on the substrate along with the AgNWs. Since residual solvents do not conduct electricity, they lowered the electrical conductivity. As the temperature increased, the probability of solvent evaporation increased, which decreased the electrical resistance of the electrode. In addition, as the temperature increased, the evaporation rate of the solvent increased, increasing the capillary force between the NWs. This increase in the capillary force increased the interconnectivity between the NWs in the network, improving the electrical conductivity. The AgNW-exposed electrode dried at room temperature showed a higher sheet resistance than that of the electrode obtained by thermal drying, which was approximately 85 Ω/sq, regardless of the drying time (Figure 4b). Since the room temperature was lower than the boiling point of the solvent, the residual solvent would remain regardless of the drying duration, resulting in a high sheet resistance. In addition, as the solvent evaporated gradually, the capillary force decreased to a level where it is insufficient to increase the interconnectivity between the NWs. Therefore, the electrodes obtained by this method exhibited the highest sheet resistance. The AgNW electrode dried by the vacuum method showed a sheet resistance of approximately 48 Ω/sq, regardless of the drying time, which was similar to that of the electrode thermally dried at 120 °C (Figure 4c). Regarding vacuum drying, there was no thermal driving force for solvent evaporation since the process proceeded at room temperature. However, because the drying pressure was low, the boiling point of the solvent was lowered, and the solvent could be easily and rapidly evaporated. Therefore, there was no residual solvent in the electrode, and a sufficient capillary force was generated during the drying process. The sufficient capillary force increased the interconnectivity between the NWs, thereby enhancing the electrical conductivity, similar to the result obtained for the sufficiently thermally dried electrode. In addition, the resistance was independent of the drying time, indicating that the solvent evaporation under vacuum drying was completed within 5 min. Next, although the AgNW networks on the substrate exhibited different resistances depending on the drying method, when they were embedded in the polymer, the AgNW-embedded electrodes showed similar resistance, regardless of the drying method and time, as shown in Figure 4d–f. Specifically, the thermally dried electrode and room-temperature-dried electrodes showed sheet resistances of approximately 32 and 35 Ω/sq, respectively. Vacuum drying led to an electrode with a sheet resistance of 33 Ω/sq. The AgNW-embedded electrode was fabricated by coating the polymer on the AgNW network of the substrate, followed by curing under UV light. This induced a shrinkage of the polymer and generated capillary force between the NWs. As mentioned above, the capillary force improved the interconnectivity between the NWs and decreased the electrical resistance. The similar resistances exhibited for all of the drying methods indicate that the capillary force generated by the polymer shrinkage was sufficient to achieve the lowest electrical resistance and to overcome the residual solvent effect on the AgNW network. Consequently, the AgNW-exposed electrodes were able to achieve a significantly low sheet resistance for electronic -device application without thermal drying. In addition, the conductivity was improved during the polymer-curing process for the AgNW-embedded electrodes even under mild drying conditions; thus, the application of various drying methods can prevent damage to electronic devices.

### 3.3. Optical Characteristics

**Figure 5:**
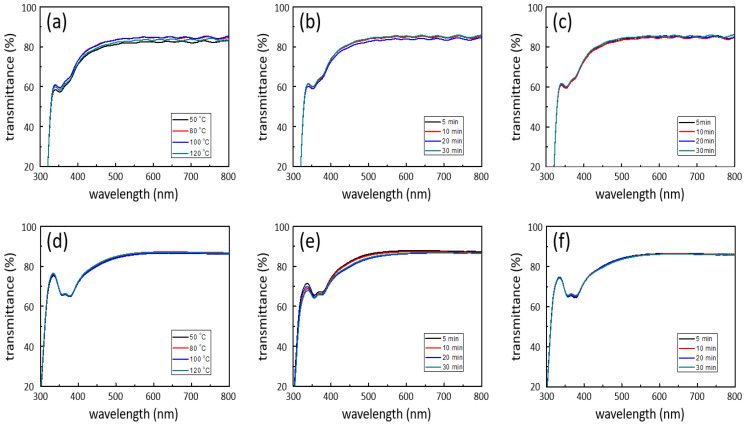
Optical transmittance of (**a**–**c**) the AgNW-exposed electrodes and (**d**–**f**) the AgNW-embedded electrodes dried by different methods against the drying temperature and time: (**a**,**d**) thermal drying, (**b**,**e**) room-temperature drying, and (**c**,**f**) vacuum drying.

UV–Vis spectroscopy was conducted to compare the optical properties of the AgNW-exposed and AgNW-embedded electrodes (Figure 5). The AgNW-exposed electrodes showed an average transmittance of approximately 83% in the visible-light region, regardless of the drying method and time. For the exposed electrode, although the drying method was different, the amount of AgNWs and the empty space between the NWs were similar for all drying conditions because the same AgNW solution was coated on the substrate under the same conditions. Consequently, the AgNW-exposed electrodes exhibited similar transmittance properties regardless of the drying method. Similarly, the AgNW-embedded electrodes showed an average transmittance of ~84% in the visible-light region for all drying methods and under all conditions. The amounts of AgNWs and empty spaces in the AgNW-embedded electrodes are similar to those in the AgNW-exposed electrodes because they were fabricated by coating and curing the polymer on AgNW-exposed substrates. Therefore, they exhibited similar transmittances. The slightly higher transmittance of the AgNW-embedded electrodes is due to the difference in the optical transmittance between PET and NOA, where the AgNW network is formed. Therefore, there was no loss in transmittance even when different drying methods were applied to prevent damage to the functional layers of the electronic devices.

### 3.4. Device Application

**Figure 6:**
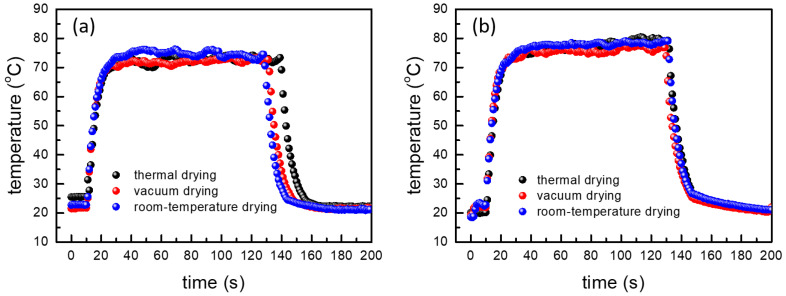
Heating characteristics of various heaters fabricated with (**a**) the AgNW-exposed and (**b**) the AgNW-embedded electrodes with various drying methods.

To demonstrate that AgNW-based electrodes with different drying methods can be used as an electrode in electronic devices, we fabricate AgNW-based heater by depositing 100 nm thick silver on AgNW-based electrodes using a thermal evaporator. Then, we measured the heating temperature under the voltage of 5 V with infrared-thermo camera system (SE/A325, Flir Systems). As shown in Figure 6, all heaters well operated and generated a comparable amount of heat. It indicates that all AgNW-based electrodes with different drying methods have the potential for electronic-device applications.

## 4. Conclusions

We investigated the effect of different drying methods and durations on the morphological, electrical, and optical properties of AgNW-based transparent electrodes. For the AgNW-exposed electrodes, thermal drying resulted in the smoothest surface, whereas room-temperature drying and vacuum drying resulted in rough surfaces. Conversely, the AgNW-embedded electrodes were formed by embedding the NWs in the polymer; thus, even though the roughness values of the AgNWs on the base substrate were different because of the different drying methods and time, the roughness values of the AgNW-embedded electrodes were similar, regardless of the drying method and time. The electrical properties of the AgNW-exposed electrodes were different because the residual amount of solvent and the magnitude of the capillary force differed depending on the drying method. Room-temperature drying, which led to the slowest solvent evaporation, resulted in the lowest conductivity, and thermal drying at 120 °C and vacuum drying resulted in similar conductivities. When these AgNW-exposed electrodes were converted into an embedded type, they exhibited similar conductivities, owing to the capillary force generated by the polymer shrinkage during the curing process and the improvement of the NW interconnectivity. The optical transmittances were similar regardless of the drying method and time for all the AgNW-based electrodes. When fabricating heaters with AgNW-exposed and AgNW-embedded electrodes with different drying methods, they showed comparable characteristics, indicating their potential for electronic-device applications. Conclusively, vacuum drying was found to be able to replace thermal drying, thereby avoiding damage to the functional layers of electronic devices. In particular, for the AgNW-embedded type, even under mild drying conditions, the embedding process of the AgNWs in the polymer resulted in high conductivity and low surface roughness. Therefore, it is possible to adopt various drying methods to prevent damage to electronic devices.

## Data Availability

Data presented in this article is available on request from the corresponding author.
